# Knowledge, attitude, influences and use of complementary and alternative medicine (CAM) among chiropractic and nursing students

**DOI:** 10.1186/s12998-017-0160-0

**Published:** 2017-10-17

**Authors:** Bruce F. Walker, Anthony Armson, Christopher Hodgetts, Angela Jacques, Fu En Chin, Garret Kow, Hyung Jin Lee, Mui Kee Wong, Anthony Wright

**Affiliations:** 1 School of Health Professions, Murdoch University, Perth, WA Australia; 2 School of Physiotherapy and Exercise Science, Curtin University, Perth, WA Australia

**Keywords:** Complementary and alternative medicine, Chiropractic, Nursing

## Abstract

**Background:**

Complementary and alternative medicine (CAM) is becoming more widely used in the community however there are differences in knowledge and attitudes among and within the various health professions. Chiropractic and nursing students represent a future generation of two health profession groups who may have differing views on CAM. The objectives of this study were to investigate the knowledge, attitudes and beliefs of nursing and chiropractic students about CAM. To investigate the factors that influence their attitudes and beliefs and their likelihood of recommending CAM; and to compare the findings between nursing and chiropractic students to determine similarities and differences.

**Methods:**

A modified and pre-tested survey including a previously validated 10-item CAM Health Belief Questionnaire (CHBQ) was administered to nursing and chiropractic students at Murdoch University. Student’s demographics were collected as well as other information regarding knowledge, attitudes, influences and use of CAM.

**Results:**

Three hundred twenty-one nursing and 227 chiropractic students responded with a 91% response rate. The CHBQ overall mean scores for nursing and chiropractic students were 47.6 and 47.4 out of possible 70 respectively, confirming positive attitudes toward CAM in both groups. Nursing and chiropractic students also demonstrated similar knowledge levels. Factors that were most influential in shaping both chiropractic and nursing students’ attitudes and beliefs towards CAM were personal experience and the influence of external peers. Nursing students would not dissuade future patients from CAM, however chiropractic students were more likely to recommend CAM to their future patients.

**Conclusions:**

Nursing and chiropractic students demonstrate relatively positive attitudes and beliefs towards CAM despite, their limited knowledge concerning CAM modalities generally.

**Electronic supplementary material:**

The online version of this article (10.1186/s12998-017-0160-0) contains supplementary material, which is available to authorized users.

## Background

Complementary and alternative medicine practices (CAM) are often classified into broad categories, such as body and mind medicine, natural products, manipulative and body-based therapies [1]. The National Institute of Complementary Medicine states that CAM, “is a broad domain of healing resources that encompasses all health systems, modalities, and practices and their accompanying theories and beliefs, other than those intrinsic to the politically dominant health system of a particular society or culture in a given historical period” [1]. The range of CAM treatments includes naturopathy, acupuncture, massage therapy, yoga, herbal medicine, chiropractic, and homeopathy [1]. Up to 69.8% of Australians have used at least one form of CAM, and 44.1% have visited a CAM practitioner in the previous 12 months [2].

The increased use of CAM among the general community has been attributed to the increased availability of information on the internet, contacts with other cultures that traditionally use CAM, the view that CAM is safer and less expensive than conventional medications and a growing recognition that many factors contribute to health and well-being [3].

Personal experience, faculty attitudes and family background ranked amongst the most common factors influencing pharmacy students’ attitudes towards CAM and their likelihood of recommending CAM therapies to future patients [4]. More than half of a cohort of pharmacy students had changes in attitudes and beliefs toward CAM, and an increased likelihood of recommending CAM therapies in future practice when they completed a course on CAM therapies [4].

Although the use of CAM therapies has been increasing in recent years, the debate about the clinical efficacy of these therapies has been controversial amongst many medical professionals. This has been largely due to a lack of scientific data surrounding many CAM therapies, compounded by variations in knowledge level and appreciation of the existing evidence about CAM amongst different healthcare professionals.

In the United States osteopathic medical students exhibit a positive attitude toward CAM with 83% reporting self-use of at least 1 CAM modality [5]. Older students were more likely than younger students to use a larger number of CAM modalities [5]. Female osteopathic medical students had more positive attitudes towards CAM therapies compared to their male counterparts and they were more likely to recommend CAM therapies to their patients [5].

A study conducted in 10 pharmacy schools in the United States demonstrated that the majority of students agreed that CAM knowledge would be needed in future pharmacy practice, however they did not necessarily possess that knowledge at the time of questioning. Students also agreed that there are limitations to conventional medicine and that patient’s values and beliefs should be integrated into the patient care process. Furthermore, the study concluded that female students and those who had previous experience with CAM exhibited a more favorable attitude towards CAM [6].

Acute care nurses in the United States reported an increase in patients seeking CAM therapies to cope with pain, particularly when they experienced inadequate pain relief from mainstream medicine [7]. Approximately half (51%) of the nurses surveyed demonstrated poor baseline information regarding CAM and even fewer (47%) were able to accurately describe or define CAM and CAM terminology [7]. This was linked to an inability to educate their patients on the different CAM therapies available and those that may be beneficial in pain management. Nevertheless, attitudes towards CAM were positive with the majority agreed that patients have a right to incorporate CAM therapies into their conventional medical treatments. The nurses also believed that the use of CAM therapies by patients should be disclosed [7].

A further survey of nursing students and staff reinforced these findings that clinical care ought to incorporate the best of CAM therapies and highlighted a positive attitude towards incorporating CAM into nursing practice. However, this study also highlighted the lack of education amongst nurses in relation to CAM therapies [8].

There does not appear to be any previous research on chiropractic students’ or Australian nursing students’ knowledge and attitudes toward CAM.

It seems important that as healthcare providers interact with patients seeking CAM in an age of patient centered care they are able to give patients advice and potentially options (where possible) for the management of disease or pain, and this may involve consideration of CAM therapies. Healthcare providers also need to be able to educate patients regarding the evidence for CAM therapies, their prognosis, adverse events and contraindications. To be able to accomplish this, healthcare providers, including chiropractors and nurses, should have a basic knowledge of other treatment options such as CAM.

In summary, there is limited research regarding knowledge, attitudes, influences and use of complementary and alternative medicine among Australian chiropractic and nursing students. The aims of this study were therefore to:

1. evaluate the knowledge of nursing and chiropractic students about CAM.
2. determine their attitudes and beliefs regarding the use of CAM.
3. determine whether or not chiropractic and nursing students would recommend CAM to their future patients. For chiropractors this means other than their own interventions.
4. investigate the factors that influence their attitudes and beliefs and their likelihood of using or recommending CAM to future patients.
5. compare the findings between nursing and chiropractic students and determine similarities and differences.

## Methods

### Participants

We recruited chiropractic and nursing students from Murdoch University in Perth, Western Australia aged 18 and over from all years of their courses.

### Recruitment

We contacted appropriate unit coordinators from each year group and asked if our team could distribute an information letter concerning the research and address students about the research. We also asked for permission to administer the survey during teaching times that suited the Unit Coordinators.

The surveys were completed in an anonymous manner, no student was obliged to participate in the study and there were no negative consequences for non-participation. No unit coordinators or teaching staff were involved directly in recruiting participants. Approval for the study was provided by the Murdoch University Human Research Ethics Committee, number 2016/137. Permission to undertake the survey was granted by the Heads of Nursing and Chiropractic at Murdoch University.

### Survey instrument

A survey was constructed based on questions utilised in previous studies [9, 10]. The survey was designed to measure students’ knowledge, attitudes and beliefs about complementary and alternative medicine (CAM) and the factors that had influenced them (Additional file 1). The instrument was trialled in a pilot study before finalisation of its content and distribution to students.

Initially, participants were requested to record if they were currently enrolled in the chiropractic or nursing course, their current academic year, their gender and age. In order to assess students’ knowledge of CAM, the survey asked students to self-rate their knowledge of 15 different CAM modalities on a four-point Likert type scale (‘good knowledge’, ‘some knowledge’, ‘aware’ or ‘unaware’) [9].

A previously validated 10-item CAM Health Belief Questionnaire (CHBQ) was also incorporated into the survey instrument. This was used to evaluate students’ attitudes and beliefs towards CAM. The CHBQ has been tested and found to be both a reliable and valid instrument for measuring attitudes/beliefs towards CAM [10]. The 10 items are framed in a seven- point Likert-type rating scale format (where 1 = “Absolutely Disagree,” and 7 = “Absolutely Agree”) with item 6, 7 and 8 reverse scored.

To determine factors that influenced students’ attitudes, a modified questionnaire from previous research was employed [6]. Students were given a list of different factors that may or may not have had an influence over their attitudes towards CAM and they were asked to rate each factor on a Likert- type scale (1-7). Highly influential responses were scored as a 7 and not at all influential responses were scored as 1. Students were also required to rate their likelihood of recommending CAM to future patients using a similar 7-point scale.

### Sample size

We sought to recruit a maximum convenience sample of students. In the case of chiropractic students it was estimated that at the time of data collection a maximum of 175 would participate and for nursing students it was estimated that a maximum of 350 would participate. These numbers are comparable to the numbers participating in previous surveys on the same topic [5, 7, 9–11].

### Data analysis

Descriptive statistics were compiled on student course, year of study, age and gender. Response rate was calculated using a numerator which was the number of students who agreed to complete the survey in the classroom on the day of survey administration. The denominator was the overall number of students present who were invited to participate.

Continuous variables including Likert scale questions were summarised using means and standard deviations and categorical data were summarised using frequency distributions. Univariate differences between student groups were analysed using independent t-tests for continuous data and chi squared or Fisher exact tests, as appropriate, for categorical variables. Data analysis was done using IBM SPSS v24.0 (Armonk, NY). *P* values <0.05 were considered statistically significant.

## Results

A total of 548 nursing and chiropractic students participated in the study in the second half of 2016. There were 321 nursing students and 227 chiropractic students which exceeds the estimated convenience sample size. Of those offered the survey instrument 5 nursing students and 2 chiropractic students declined to participate (response rate is 98.44 and 99.12 respectively). The nursing and chiropractic students who responded to the survey had a mean age of 27.7 years (SD:9.0) and 22.3 years (SD:3.9) respectively. Of those who participated in the study, 93.3% of the nursing students and 56.4% of the chiropractic students were female. The total number of nursing and chiropractic student respondents by course year was as follows: year 1, 189 students; year 2, 198 students; year 3, 96 students; year 4, 28 students (chiropractic only); and year 5, 36 students (chiropractic only).

Table 1 shows the mean score for knowledge of different CAM modalities in chiropractic and nursing students ranked from highest to lowest. Table 2 illustrates the comparative differences in knowledge of CAM modalities by nursing and chiropractic survey respondents with statistical differences in awareness of CAM modalities between nursing and chiropractic students seen in massage, prayer, chiropractic, Reiki, osteopathy and hypnosis (*p* < 0.001).

**Table 1:** Knowledge of CAM (Comparison between Chiropractic and Nursing students)

CAM –chiropractic students	Mean[0-3] (SD)	CAM –nursing students	Mean[0-3] (SD)
Chiropractic	2.59 (0.57)	Meditation / Relaxation	1.91 (0.76)
Massage	2.06 (0.80)	Yoga	1.89 (0.80)
Yoga	1.84 (0.78)	Nutritional therapy (incl. Herbal medicine, supplements)	1.74 (0.68)
Nutritional therapy (incl. Herbal medicine, supplements)	1.82 (0.69)	Massage	1.72 (0.74)
Meditation / Relaxation	1.74 (0.72)	Spirituality / Prayer	1.60 (0.93)
Acupuncture	1.54 (0.79)	Chiropractic	1.52 (0.82)
Naturopathy	1.20 (0.88)	Acupuncture	1.39 (0.74)
Spirituality / Prayer	1.16 (0.92)	Naturopathy	1.22 (0.80)
Osteopathy	1.15 (0.77)	Hypnosis	1.13 (0.80)
Homeopathy	0.96 (0.81)	Therapeutic Touch / Reiki	1.11 (0.89)
Hypnosis	0.89 (0.72)	Homeopathy	0.96 (0.81)
Therapeutic Touch / Reiki	0.83 (0.85)	Tai Chi / Qi Gong	0.79 (0.80)
Tai Chi / Qi Gong	0.78 (0.73)	Osteopathy	0.71 (0.74)
Biofeedback	0.45 (0.69)	Ayurveda	0.40 (0.75)
Ayurveda	0.34 (0.68)	Biofeedback	0.36 (0.65)

**Table 2:** Mean difference between Chiro and Nursing students

Modality	Mean [(SE) of difference	95%CI Mean Diff	*P* value
1. Nutrition	0.084 (0.1)	(−0.033, 0.202)	0.160
2. Massage	0.342 (0.1)	(0.212, 0.472)	<0.001
3. Spirituality / Prayer	−0.437 (0.1)	(−0.596, −0.278)	<0.001
4. Chiropractic	1.073 (0.1)	(0.956, 1.19)	<0.001
5. Homeopathy	0.003 (0.1)	(−0.136, 0.141)	0.97
6. Naturopathy	−0.017 (0.1)	(−0.159, 0.126)	0.819
7. Acupuncture	0.152 (0.1)	(0.022, 0.282)	0.022
8. Meditation	−0.167 (0.1)	(−0.294, −0.039)	0.010
9. Reiki	−0.285 (0.1)	(−0.434, −0.136)	<0.001
10. Tai Chi	−0.02 (0.1)	(−0.152, 0.112)	0.77
11. Osteopathy	0.442 (0.1)	(0.313, 0.571)	<0.001
12. Hypnosis	−0.247 (0.1)	(−0.376, −0.118)	<0.001
13. Ayurveda	−0.058 (0.1)	(−0.181, 0.066)	0.359
14. Biofeedback	0.089 (0.1)	(−0.024, 0.202)	0.124
15. Yoga	−0.056 (0.1)	(−0.192, 0.079)	0.412

Only 8 therapies (Modalities 2, 3, 4, 7, 8, 9, 11, 12) are statistically different, *p* < 0.05

The overall mean scores of knowledge of different CAM therapies for nursing and chiropractic students were 1.23 (SD: 0.47) and 1.29 (SD: 0.41) respectively. There were no significant differences between the student groups in overall knowledge score. In contrast to nursing students, chiropractic students have more knowledge of osteopathy (*p* < 0.001), whereas nursing students have more knowledge of spirituality than chiropractic students (*p* < 0.001). There were no significant differences between nursing and chiropractic students in nutrition, homeopathy, Tai chi, Ayurveda, biofeedback and yoga modalities. However, among all modalities listed, nutrition, massage, chiropractic, meditation and yoga were most known by respondents.

For the CAM Health Belief Questionnaire (Tables 3 and 4) overall grand mean scores for nursing and chiropractic students were 47.60 (SD: 8.18) and 47.35 (SD: 8.77) respectively, exceeding a hypothetical scale midpoint of 40 confirming positive beliefs/attitudes toward CAM in both groups [10]. In contrast to chiropractic students, nursing students agreed more strongly that patient’s expectations, health beliefs, and values should be integrated into the patient care process (mean = 6.13 vs. 5.91) There was some agreement that CAM is a threat to public health (Table 5).

**Table 3:** CAM Beliefs of Chiropractic students

*Statement*	Mean[1–7], SD
A patient’s expectations, health beliefs and values should be integrated into the patient care process.	5.91 (1.36)
Complementary and alternative therapies include ideas and methods from which conventional medicine could benefit.	5.17 (1.42)
The body is essentially self-healing and the task of a health care provider is to assist in the healing process	5.04 (1.4)
A patient’s symptoms should be regarded as a manifestation of a general imbalance or dysfunction affecting the whole body	4.48 (1.54)
Most complementary and alternative therapies stimulate the body’s natural therapeutic powers.	4.3 (1.57)
Treatments not tested in a scientifically recognized manner should be discouraged.	3.92 (1.84)
Health and disease are a reflection of balance between positive life-enhancing forces and negative destructive forces.	3.78 (1.78)
The physical and mental health is maintained by an underlying energy or vital force.	3.76 (1.72)
Effects of complementary and alternative therapies are usually the result of a placebo effect	2.94 (1.45)

**Table 4:** CAM Beliefs of Nursing students

*Statement*	Mean[1–7], SD
A patient’s expectations, health beliefs and values should be integrated into the patient care process.	6.13 (1.27)
The body is essentially self-healing and the task of a health care provider is to assist in the healing process	4.96 (1.56)
A patient’s symptoms should be regarded as a manifestation of a general imbalance or dysfunction affecting the whole body	4.78 (1.51)
Complementary and alternative therapies include ideas and methods from which conventional medicine could benefit.	4.65 (1.32)
Most complementary and alternative therapies stimulate the body’s natural therapeutic powers.	4.41 (1.37)
Health and disease are a reflection of balance between positive life-enhancing forces and negative destructive forces.	4.39 (1.62)
The physical and mental health is maintained by an underlying energy or vital force.	4.33 (1.64)
Treatments not tested in a scientifically recognized manner should be discouraged.	3.72 (1.73)
Effects of complementary and alternative therapies are usually the result of a placebo effect	3.56 (1.47)
Complementary and alternative therapies are a threat to public health	2.52 (1.66)

**Table 5:** Mean difference between Chiropractic and Nursing students regarding CAM beliefs

	Mean (SE) of difference	95% CI Mean Diff	*P* value
1.The physical and mental health is maintained by an underlying energy or vital force.	−0.565 (0.1)	(−0.85, −0.28)	<0.001
2. Health and disease are a reflection of balance between positive life-enhancing forces and negative destructive forces.	−0.611 (0.1)	(−0.9, −0.322)	<0.001
3. The body is essentially self-healing and the task of a health care provider is to assist in the healing process.^a^	0.07 (0.1)	(−0.184, 0.324)	0.589
4. A patient’s symptoms should be regarded as a manifestation of a general imbalance or dysfunction affecting the whole body	−0.296 (0.1)	(−0.555, −0.037)	0.025
5. A patient’s expectations, health beliefs and values should be integrated into the patient care process.	−0.232 (0.1)	(−0.454, −0.01)	0.041
6. Complementary and alternative therapies are a threat to public health.^b^	−0.341 (0.1)	(−0.607, −0.075)	0.012
7. Treatments not tested scientifically discouraged.^ab^	0.204 (0.2)	(−0.099, 0.507)	0.187
8. Effects of complementary and alternative therapies are usually the result of a placebo effect.^b^	−0.624 (0.1)	(−0.874, −0.374)	<0.001
9. Complementary and alternative therapies include ideas and methods from which conventional medicine could benefit.	0.508 (0.1)	(0.275, 0.742)	<0.001
10. Most complementary and alternative therapies stimulate the body’s natural therapeutic powers.^a^	−0.135 (0.1)	(−0.385, 0.114)	0.288

^a^Statement with no significant differences

^b^Item responses were reverse scored so a higher value indicated greater endorsement. Responses were based on a Likert-type scale ranging from 1 = absolutely disagree and 7 = absolutely agree

**Fig. 1:**
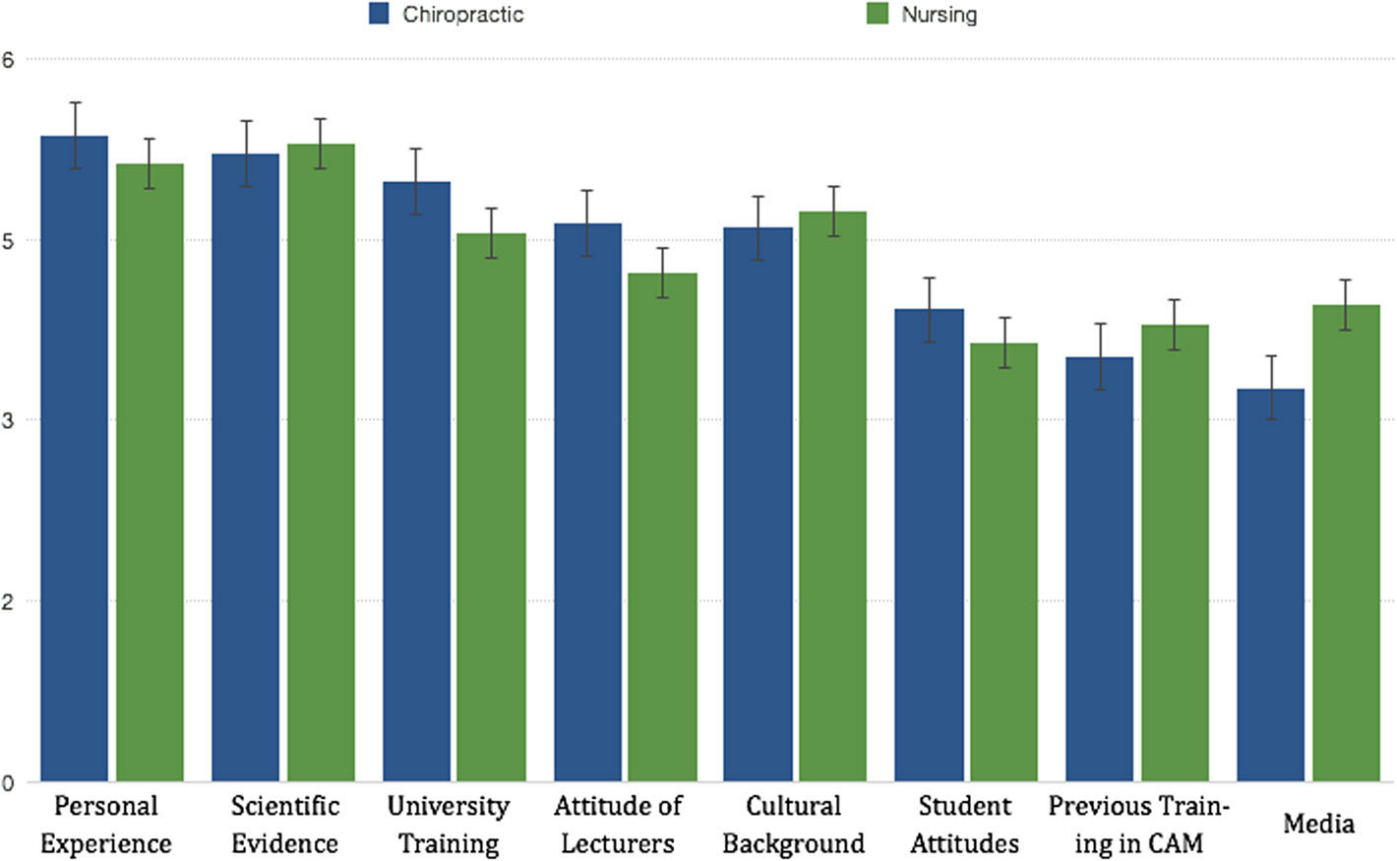
Factors that influenced respondents’ belief in CAM

Among the factors that influenced respondents’ beliefs in relation to CAM, personal experience was the main factor for both nursing (mean = 5.37) and chiropractic students (mean = 5.13). Chiropractic students were more influenced by university training (mean [SD] 4.99, 1.6 vs. 4.56, [1.8]), attitudes of lecturers (mean [SD]4.64, 1.6 vs. 4.23, [1.8]) and opinions of practitioners (mean [SD] 5.22, 1.4 vs. 5.03, [1.6]) compared to nursing students (*p* < 0.05). Moreover, media had a greater impact on nursing students (mean 3.96, SD 1.6) than on chiropractic students (mean 3.27, SD 1.6) p < 0.05 (Fig. 1).

Regarding recommending CAM therapies to patients, chiropractic students (mean = 5.21) were more likely to recommend CAM compared to nursing students (mean [SD] 4.52, 1.5) (p < 0.001).

## Discussion

This study was designed to assess the knowledge, attitudes and beliefs of chiropractic and nursing students towards CAM. We also looked at factors that influenced their views and the likelihood of recommending CAM to their future patients. Nursing students demonstrated limited knowledge regarding CAM with mean self-rated knowledge of 9 out of the 15 CAM modalities falling between “aware” and “some knowledge” and 5 out of 15 modalities falling between “not aware” and “aware”. Another study measuring nurse’s knowledge and attitudes towards CAM^7^ revealed similar results in that nurses demonstrated poor baseline knowledge of CAM modalities. Similarly chiropractic students’ self-rated knowledge was also limited with 8 of the CAM modalities (other than chiropractic) falling between “aware” and “some knowledge” and 6 out of 15 modalities falling between “not aware” and “aware”. As a whole, their levels of knowledge were not significantly different and it can be argued that for both groups their knowledge could be enhanced. Despite the limited knowledge nursing and chiropractic students had regarding CAM, both groups still demonstrated positive attitudes towards CAM. However, it is noted there was a minority of chiropractic students who agreed with the statement “complementary and alternative therapies are a threat to public health” and would not recommend CAM to future patients despite chiropractic generally being categorized as a CAM profession. This counter-intuitive finding may reflect some chiropractic students’ views that they are more part of mainstream medicine or allied health rather than CAM. These findings are comparable to previous studies on medical students which also indicated that despite having limited knowledge regarding CAM, medical students still reported positive attitudes towards CAM and would welcome education on CAM in their curriculum [12].

When questioned about the belief that “A patient’s expectations, health beliefs and values should be integrated into the patient care process” both chiropractic and nursing students gave similar responses to this statement with strong agreement from both groups. This agreement between chiropractic and nursing students on patient centred care is consistent with previous studies involving nurses and pharmacy students [6, 7].

Among nursing and chiropractic students the main factors that influenced their attitudes and beliefs towards CAM were the same, with personal experience with CAM, the opinions of chiropractors or nurses (respectively) outside their current university training and scientific evidence ranking among the top three most influential factors respectively shaping their attitudes and beliefs about CAM. A previous study also showed a similar result for pharmacy students with personal experience and university training being factors that heavily influenced them [6]. It was interesting to note that personal experience and the opinions of senior practitioners was a stronger factor than the influence of their respective lecturers. This may have positive or negative consequences depending on what influences are brought to bear on the students by external practitioners. It can be argued that scientific evidence should be the number one influence on health professional students.

For nursing students, their fellow student’s attitudes and beliefs were the least influential whereas for chiropractic students it was the media that was the least influential. Chiropractic has at times attracted controversy in the media and has been portrayed negatively [13–15]; it is possible that this may be one reason why the media was the least influential among chiropractic students while greater reliance on evidence by chiropractic students may also be another determining factor. Likewise, fellow student’s attitudes and beliefs along with the media were the least influential factors for pharmacy students [6].

With regards to recommending CAM to future patients, chiropractic students leaned more favourably towards recommending CAM than nursing students. A previous study conducted with medical students displayed comparable results to the nursing students in that the majority of students would neither persuade nor dissuade patients from using CAM [12]. The authors of this study attributed these results to the students’ lack of knowledge or confidence in CAM modalities, and this may also be a factor for the nurses in our study.

### The public health implications of this research

It has been reported that up to 72% of patients do not disclose the use of CAM to their conventional medical doctor [16]. Given that certain CAM treatments can reduce efficacy or have adverse interactions with conventional medicine [17], it is important for chiropractors as primary healthcare providers, and nurses as patient carers to be well equipped with contemporary evidence based knowledge on the interactions and effects of CAM. This knowledge is likely to enable chiropractors and nurses to offer the most effective and safest healthcare to their patients, while still providing them with multiple options for care.

### Strengths and limitations

During this study we received a high response rate from both nursing and chiropractic cohorts. Among the 548 surveys that were distributed, 543 were completed and only 5 surveys were left incomplete giving a response rate of 99.1%. Furthermore, the survey questions used in this study were pre-tested and used in previous studies, making the questionnaires more reliable in terms of their comparative usability [6, 7]. The questions used to assess attitudes and beliefs towards CAM have also been validated and tested for feasibility [10].

As the surveys were only distributed in a single institution the generalisability of the results to other nursing and chiropractic students is limited. In addition, this study was limited to only two health professions. The results obtained from this study may differ across different health professions.

In addition, any differences between the nursing and chiropractic student groups may be explained, at least in part, by differences in their curricula as well as differences in the practice roles for which they are being prepared.

Future research should concentrate on expanding the study to other nursing and chiropractic programs and increasing the number of health professions surveyed to allow for a more in depth evaluation of similarities and differences among different health professions. It would be also useful to survey chiropractic students about their identity as health professionals and whether they regard themselves as CAM or allied health practitioners. Finally, studies comparing healthcare students and practicing healthcare professionals may also provide another avenue for future research.

## Conclusions

This study provides information regarding knowledge, attitudes and beliefs, factors that influence and the likelihood of recommending CAM to future patients among two different health professions, chiropractic and nursing students. The results in this study demonstrate relatively positive attitudes and beliefs towards CAM from both nursing and chiropractic students, despite them having limited knowledge concerning different CAM modalities. Personal experience and the influence of external practitioners were noted to be the most influential factors in shaping both chiropractic and nursing students’ CAM attitudes and beliefs, and although nursing students would not dissuade future patients from CAM, chiropractic students were more likely to recommend CAM to their future patients.

## Additional file

Additional file 1: Survey instrument. (DOCX 15 kb)
