# Supplementary material for: Knowledge, attitude, influences and use of complementary and alternative medicine (CAM) among chiropractic and nursing students
Source: Chiropr Man Therap. 2017 Oct 17;25:29. doi: 10.1186/s12998-017-0160-0 (PMC5644107; doi:10.1186/s12998-017-0160-0)
Supplement: Additional file 1: — Survey instrument. (DOCX 15 kb) [file 12998_2017_160_MOESM1_ESM.docx]

**Additional file 1. Survey instrument**

School of Health Professions

Office of Associate Professor Bruce Walker AM

| 90 South Street, Murdoch  Western Australia 6150  Telephone: +61 8 9360 1297 |
| --- |

*** Completion of this anonymous survey indicates your consent to participate. ***

For each of the following 15 CAM modalities please **tick the box** [ ✔ ] representing the statement that most accurately describes your current knowledge concerning them

| **Modality** | **Good Knowledge** | **Some Knowledge** | **Aware** | **Not Aware** |
| --- | --- | --- | --- | --- |
| Nutritional therapy (incl. herbal medicine, supplements) |  |  |  |  |
| Massage |  |  |  |  |
| Spirituality / Prayer |  |  |  |  |
| Chiropractic |  |  |  |  |
| Homeopathy |  |  |  |  |
| Naturopathy |  |  |  |  |
| Acupuncture |  |  |  |  |
| Meditation / Relaxation |  |  |  |  |
| Therapeutic Touch / Reiki |  |  |  |  |
| Tai Chi / Qi Gong |  |  |  |  |
| Osteopathy |  |  |  |  |
| Hypnosis |  |  |  |  |
| Ayurveda |  |  |  |  |
| Biofeedback |  |  |  |  |
| Yoga |  |  |  |  |
| Others (Please specify)  ________________________ |  |  |  |  |

Please read and tick [ ✔ ] to the following statements according to your beliefs, using numbers 1-7 where **1 is absolutely disagree** and **7 is absolutely agree**.

01. The physical and mental health is maintained by an underlying energy or vital force.

Absolutely Disagree 1 2 3 4 5 6 7 Absolutely Agree

02. Health and disease are a reflection of balance between positive life-enhancing forces and negative destructive forces.

Absolutely Disagree 1 2 3 4 5 6 7 Absolutely Agree

03. The body is essentially self-healing and the task of a health care provider is to assist in the healing process.

Absolutely Disagree 1 2 3 4 5 6 7 Absolutely Agree

04. A patient's symptoms should be regarded as a manifestation of a general imbalance or dysfunction affecting the whole body.

Absolutely Disagree 1 2 3 4 5 6 7 Absolutely Agree

05. A patient's expectations, health beliefs and values should be integrated into the patient care process.

Absolutely Disagree 1 2 3 4 5 6 7 Absolutely Agree

06. Complementary and alternative therapies are a threat to public health.

Absolutely Disagree 1 2 3 4 5 6 7 Absolutely Agree

07. Treatments not tested in a scientifically recognized manner should be discouraged.

Absolutely Disagree 1 2 3 4 5 6 7 Absolutely Agree

08. Effects of complementary and alternative therapies are usually the result of a placebo effect.

Absolutely Disagree 1 2 3 4 5 6 7 Absolutely Agree

09. Complementary and alternative therapies include ideas and methods from which conventional medicine could benefit.

Absolutely Disagree 1 2 3 4 5 6 7 Absolutely Agree

10. Most complementary and alternative therapies stimulate the body's natural therapeutic powers.

Absolutely Disagree 1 2 3 4 5 6 7 Absolutely Agree

For the following 8 factors please indicate if they have influenced your attitudes and beliefs regarding CAM using numbers 1-7 where **1 is not at all influential** and **7 is highly influential

The following have influenced my attitudes and beliefs** regarding CAM: please **tick the box** [ ✔ ]

1. Personal Experience:

Not At All Influential 1 2 3 4 5 6 7 Highly Influential

2. University Training:

Not At All Influential 1 2 3 4 5 6 7 Highly Influential

3. Attitudes of Lecturers & Tutors:

Not At All Influential 1 2 3 4 5 6 7 Highly Influential

4. My cultural Background (incl. Family influences):

Not At All Influential 1 2 3 4 5 6 7 Highly Influential

5. Fellow Students Attitudes:

Not At All Influential 1 2 3 4 5 6 7 Highly Influential

6. Media (incl. Social Media, TV, Internet):

Not At All Influential 1 2 3 4 5 6 7 Highly Influential

7. Previous Training / Course in CAM:

Not At All Influential 1 2 3 4 5 6 7 Highly Influential

8. Scientific Evidence:

Not At All Influential 1 2 3 4 5 6 7 Highly Influential

**9n. If you are a nursing student please answer this question:**
The opinions of a nurse or nurses I know:

Not At All Influential 1 2 3 4 5 6 7 Highly Influential

**9c. If you are a chiropractic student please answer this question:**
The opinions of chiropractor(s) external to the course of study at Murdoch:

Not At All Influential 1 2 3 4 5 6 7 Highly Influential

What is the likelihood of you recommending CAM to future patients?

Unlikely 1 2 3 4 5 6 7 Very likely

**Background Information**

I am currently enrolled in: Chiropractic Nursing

Current Year in Course: Year 1 Year 2 Year 3 Year 4 Year 5

Gender: Female Male

Your age: _____ (years)
